# Validation of Fitbit Charge 2 Sleep and Heart Rate Estimates Against Polysomnographic Measures in Shift Workers: Naturalistic Study

**DOI:** 10.2196/26476

**Published:** 2021-10-05

**Authors:** Benjamin Stucky, Ian Clark, Yasmine Azza, Walter Karlen, Peter Achermann, Birgit Kleim, Hans-Peter Landolt

**Affiliations:** 1 Institute of Pharmacology and Toxicology University of Zurich Zurich Switzerland; 2 Sleep & Health Zurich, University Center of Competence University of Zurich Switzerland; 3 Department of Experimental Psychopathology and Psychotherapy University of Zurich Zurich Switzerland; 4 Department of Psychiatry, Psychotherapy and Psychosomatics University Hospital for Psychiatry University of Zurich Zurich Switzerland; 5 Department of Psychiatry and Psychotherapy Translational Psychiatry Unit University of Lubeck Lubeck Germany; 6 Mobile Health Systems Lab Department of Health Sciences and Technology ETH Zurich Zurich Switzerland; 7 The Key Institute for Brain-Mind Research, Department of Psychiatry, Psychotherapy and Psychosomatics University Hospital for Psychiatry University of Zurich Zurich Switzerland

**Keywords:** wearables, actigraphy, polysomnography, validation, multisensory, mobile phone

## Abstract

**Background:**

Multisensor fitness trackers offer the ability to longitudinally estimate sleep quality in a home environment with the potential to outperform traditional actigraphy. To benefit from these new tools for objectively assessing sleep for clinical and research purposes, multisensor wearable devices require careful validation against the gold standard of sleep polysomnography (PSG). Naturalistic studies favor validation.

**Objective:**

This study aims to validate the Fitbit Charge 2 against portable home PSG in a shift-work population composed of 59 first responder police officers and paramedics undergoing shift work.

**Methods:**

A reliable comparison between the two measurements was ensured through the data-driven alignment of a PSG and Fitbit time series that was recorded at night. Epoch-by-epoch analyses and Bland-Altman plots were used to assess sensitivity, specificity, accuracy, the Matthews correlation coefficient, bias, and limits of agreement.

**Results:**

Sleep onset and offset, total sleep time, and the durations of rapid eye movement (REM) sleep and non–rapid-eye movement sleep stages N1+N2 and N3 displayed unbiased estimates with nonnegligible limits of agreement. In contrast, the proprietary Fitbit algorithm overestimated REM sleep latency by 29.4 minutes and wakefulness after sleep onset (WASO) by 37.1 minutes. Epoch-by-epoch analyses indicated better specificity than sensitivity, with higher accuracies for WASO (0.82) and REM sleep (0.86) than those for N1+N2 (0.55) and N3 (0.78) sleep. Fitbit heart rate (HR) displayed a small underestimation of 0.9 beats per minute (bpm) and a limited capability to capture sudden HR changes because of the lower time resolution compared to that of PSG. The underestimation was smaller in N2, N3, and REM sleep (0.6-0.7 bpm) than in N1 sleep (1.2 bpm) and wakefulness (1.9 bpm), indicating a state-specific bias. Finally, Fitbit suggested a distribution of all sleep episode durations that was different from that derived from PSG and showed nonbiological discontinuities, indicating the potential limitations of the staging algorithm.

**Conclusions:**

We conclude that by following careful data processing processes, the Fitbit Charge 2 can provide reasonably accurate mean values of sleep and HR estimates in shift workers under naturalistic conditions. Nevertheless, the generally wide limits of agreement hamper the precision of quantifying individual sleep episodes. The value of this consumer-grade multisensor wearable in terms of tackling clinical and research questions could be enhanced with open-source algorithms, raw data access, and the ability to blind participants to their own sleep data.

## Introduction

Highly sensitive and precise instruments are necessary for the accurate measurement of sleep in healthy and clinical populations. Polysomnography (PSG), the prevailing gold standard in clinical and research settings [1], reliably reflects the physiological processes underlying sleep with high temporal resolution [2]. The PSG recordings are conducted to capture sleep macrostructure (eg, stages and cycles) and microstructure (eg, K-complexes, spindles, and arousals), and to quantify different variables such as power density spectra of the electroencephalogram and heart rate (HR) variability, to estimate an individual’s sleep quality and health. Despite the many strengths of PSG, attendant disadvantages include high cost, the need for personnel trained in technical aspects and interpretation of data, and the highly technical recording system itself, which usually necessitates a dedicated sleep laboratory, although ambulatory systems also exist [3]. Inexpensive, practical, and portable alternatives that are equally accurate and reliable as PSG in measuring sleep would be welcome for clinicians and researchers.

Currently, the only validated and United States Food and Drug Administration–approved alternative to PSG in ambulatory settings is actigraphy [4]. Actigraphy measures movement using a multiaxis accelerometer in a device resembling a wristwatch, sometimes accompanied by an embedded light sensor. Actigraphy captures rest-activity behaviors such as sleep habits, bedrest, rise times, and light exposure [5,6]. The basic assumption of actigraphy is that motion implies wakefulness, whereas no motion implies sleep. Fully disclosed algorithms [7,8] are used to compute sleep variables with some precision, but performance compared with PSG varies because of the inherent limitation in discriminating sleep from waking that is not accompanied by movement [3,4]. Actigraphy is a dedicated scientific instrument in clinical and research contexts and depends on specialists for setting up and interpreting data [9].

Recently, there has been greater acceptance, but also controversy, among the scientific community about using commercially available wearable devices such as fitness trackers in research [10]. Fitness trackers are multisensor, consumer-grade devices that represent a cost-efficient, practical, and convenient means of objectively collecting rest-activity data longitudinally under ambulatory conditions [4]. Fitbit is a market leader [11], and efforts have been made to validate its devices, such as the Fitbit Charge 2, against PSG [4,12-15] and the portable single-channel electroencephalogram sleep scope device [16]. Such devices not only rely on movement but also measure HR via photoplethysmography. Changes in the activity of the autonomic nervous system regulating HR are coupled to changes in electroencephalogram patterns [17,18], and various HR measures are correlated with electroencephalogram-defined sleep states [19]. These relationships potentially permit a multisensor fitness tracker to estimate an array of sleep variables above and beyond that of conventional actigraphy [20-22].

A recent laboratory-based validation study suggested that the proprietary algorithm of Fitbit Charge 2 (Fitbit Inc) to estimate different sleep variables performed reasonably well [3]. More specifically, the device displayed a 9-minute overestimation of total sleep time (TST), whereas sleep onset (S_on_) latency was underestimated by 4 minutes. Furthermore, Fitbit’s *light* stage was overestimated by 34 minutes, and Fitbit’s *deep* sleep stage, assumed to be equivalent to the N3 sleep stage, was underestimated by 24 minutes compared with the PSG-derived sleep stages N1+N2 and N3, respectively. No bias was observed in wakefulness after S_on_ (WASO) or the duration of rapid eye movement (REM) sleep stage. Findings in patients with periodic limb movements during sleep revealed comparable results [3]. In contrast, a study in patients with obstructive sleep apnea contradicted the unbiasedness of WASO for 2 Fitbit devices, Fitbit Charge 2 and Fitbit Alta HR. Both devices underestimated WASO, possibly indicating variable performance in different clinical populations [23]. Other work performed at participants’ homes compared Fitbit Charge 2 with a portable single-channel electroencephalogram sleep monitor [16]. This study showed 86.9% agreement; however, there was an underestimation of TST by 12.3 minutes, of *light* sleep by 42.4 minutes, and of REM sleep by 11.6 minutes. Conversely, WASO was overestimated by 24.5 minutes and deep sleep by 39.8 minutes. These estimates also showed a large SD.

Regarding HR, a study found a moderate underestimation of 5.9 beats per minute (bpm) with Fitbit Charge 2 compared with the electrocardiogram, whereas precision for individual measurements was poor as reflected by wide limits of agreement (LoA) [24]. Another study found that this device tended to slightly overestimate HR in ranges <50 bpm (bias=0.51 bpm) and underestimate HR in ranges >80 bpm (bias=0.63 bpm) compared with the electrocardiogram [13]. The Fitbit Charge HR model displayed a general underestimation (bias=0.88 bpm) in a similar range [25].

Therefore, the findings of previous sleep and HR validation studies of Fitbit Charge 2 are rather inconsistent and warrant further research. It was previously concluded that apart from the sample population studied, inaccurate temporal synchronization between Fitbit wearables and PSG is an important challenge in some validation studies [26]. In addition, consumer-grade wearables need to be validated under naturalistic conditions and in diverse populations, as such factors may affect their performance. We attempted to validate Fitbit Charge 2 against gold-standard PSG in a healthy study sample, but one that regularly performed shift work and exhibited an elevated risk of occupational stressors, which likely interfered with and attenuated the quality of sleep. With these objectives in mind, we seek to validate the usefulness of Fitbit Charge 2 to evaluate sleep quality in first responder shift workers under naturalistic conditions, with a special focus on rigorous data preprocessing and time alignment of the data recordings.

## Methods

### Study Sample

The participants of this study were recruited from July 2017 to November 2019 by various informational media, emails, and presentations at shift change as part of a larger study investigating sleep and resilience to psychological stress and trauma. They completed 1 month of monitoring of wrist-derived rest-activity behavior with a Fitbit Charge 2 that was worn continuously by all individuals on their nondominant wrist.

The Ethics Commission of the Canton of Zurich approved (2016-01357) all study protocols and experimental procedures, and written informed consent was obtained before participation. Participants invited to participate fulfilled all inclusion criteria: aged between 18 and 65 years, BMI ≤26 (or if exceeding a BMI of 26, which is typical of very athletic participants, an absence of sleep problems, such as sleep breathing disorders, was reported), current employment in 1 of 2 participating emergency rescue stations and a police station in the greater Zurich area of Switzerland, possession of a smartphone, and German language fluency. Exclusion criteria included the presence of a neurological disorder diagnosis or head injury with the potential to affect electroencephalogram variables, reported intake of >5 alcoholic beverages per week, or if a urine drug screen (Drug Screen Multi 12-AE; Nal von Minden GmbH) revealed drug abuse. All participants were shift workers, although specific shift schedules varied among individuals by occupation, such that emergency medical rescue workers and emergency doctors worked cycles of two 12-hour days followed by two 12-hour nights, terminating in 4 free days. Police officers worked four contiguous shifts with varying individual activities and bedrest times. Data on individual shifts were not collected or analyzed. Individuals received monetary compensation for participating in the study. Participants additionally received a report on their sleep derived from their own sleep data derived from Fitbit Charge 2 and PSG. This report was explained to them by a study staff member.

Validated German translations of questionnaires administered at meetings at the start and upon completion of 1 month of monitoring were used to assess lifestyle and psychological and sleep variables. The Pittsburgh Sleep Quality Index (PSQI) [27], Posttraumatic Stress Disorder Checklist for Diagnostic and Statistical Manual of Mental Disorders, Fifth Edition [28], and the Perceived Stress Scale 10 (PSS-10) [29] were used to assess subjective sleep quality, posttraumatic stress symptoms, and stress in the past month. Cutoff scores of ≥5 on the PSQI, >31 on the Posttraumatic Stress Disorder Checklist for Diagnostic and Statistical Manual of Mental Disorders, Fifth Edition, and substantial deviations from the normative values—12.1 (SD 5.9) for men and 13.7 (SD 6.6) for women—indicate poor sleep quality [27], a probable posttraumatic stress disorder diagnosis [28], and elevated perceived stress [29], respectively. The Horne-Östberg Morningness-Eveningness Questionnaire-A Reduced Scale (rMEQ) was used to assess the participants’ preferred rest-activity behavior or *chronotype*, with higher scores indicating increased morning activity preference. Scores on the rMEQ have a range of 4-25. A previous study found that most individuals (60%; scores: 12-17) show neither a pronounced evening (20% of individuals; scores: 4-11) nor morning (20% of individuals; scores: 18-25) activity preference [30].

### Polysomnographic Recordings

A total of 62 individuals (43 emergency medical rescue workers, 16 police officers, and 3 emergency doctors), of whom 56% (35/62) were women, completed 2 nights of ambulatory PSG recordings in their homes. The PSG recordings were always made of nocturnal sleep following a day work shift and consisted of an adaptation night and then a baseline night the following evening. Individuals were free to determine their bedtime and sleep duration. The adaptation night served as a combined adaptation and screening night, whereas the baseline night provided the data analyzed in this report, with the exception of 8 individuals, whose data originated from the adaptation night because the PSG data of the baseline nights were of poor quality. The PSG data from one individual were excluded from the analyses because the data were of poor quality on both nights. Therefore, the total PSG sample consisted of 61 individuals. On 2 nights, the Fitbit Charge 2 data sets for 2 individuals were not obtained, reducing the sample to 59 individuals who had both PSG and Fitbit Charge 2 data for comparison. All PSG data were acquired using dedicated ambulatory polysomnographic amplifiers (SOMNOscreen Plus, SOMNOmedics GmbH). All electrodes and sensors for PSG recordings were applied by trained members of the research team. The overall PSG montage consisted of scalp electrode sites Fz, Cz, Pz, Oz, C3, C4, A1, and A2 applied according to the International 10-20 System [31] and electrooculogram, submental electromyogram, and electrocardiogram and grounding electrode according to the American Academy of Sleep Medicine standards [32]. The Cz electrode served as the reference during recording, and the opposite mastoid was used for the rereferenced display. The sampling rate for all the sites was 256 Hz. For recording, high-pass (0.2 Hz) and low-pass filters (128.0 Hz) were used. High-pass (0.3 Hz) and low-pass (35.0 Hz) filters in addition to a powerline filter were applied for visual sleep scoring. Sleep stages were scored visually by an experienced individual in 20-second epochs according to the American Academy of Sleep Medicine (2007) criteria.

The electrocardiogram trace in the PSG recordings was examined visually for one epoch at a time for all wake epochs before S_on_ and all epochs of sleep and wake stages after S_on_ (performed by an experienced individual). Artifacts and ectopic beats present in the electrocardiogram trace that had the potential to interfere with the quantification of interbeat intervals (IBIs), defined as the time interval between the normal R peaks of the QRS complex, were manually marked and removed before data processing and analysis.

### Fitbit Charge 2 Recordings

All participants wore the Fitbit Charge 2 continuously during the PSG recorded nights. The device records wrist activity using accelerometry and pulses via photoplethysmography. It produces two types of sleep data depending on whether certain criteria are fulfilled during data collection. These criteria are sufficient battery charge, a sleep episode >3 hours in duration, and sufficient skin contact with the photoplethysmography sensor. If these criteria are not fulfilled, then *classic* sleep data are generated, comprising *asleep*, *awake*, and *restless* variables at a 1‑minute data granularity. If these criteria are fulfilled, then *stages* data are produced, comprising *wake*, *light*, *REM*, and *deep* sleep at a 30-second data granularity. If *stages* data are obtained for a given sleep episode, then users receive two data sets, that is, (1) sleep data, which is composed of stages, and (2) wake data, which is composed exclusively of wake episodes <30 seconds. Both data sets are present in a single JSON file for a given data collection date. However, there were also wake episodes contained within the sleep data set. This data structure is especially relevant for researchers who wish to extract entire hypnogram data and information not provided by Fitbit, such as REM sleep latency (REML). The variable WASO was created in this study by merging these two data sets contained within the *stages* data type output. The Fitbit sleep staging algorithm occasionally scores the first stage after S_on_ and the last stage before sleep offset (S_off_) as *wake*. This runs counter to the intuitive definition of S_on_ and S_off_ as the first occurrence of sleep and the last occurrence of sleep, respectively.

We manually omitted such bordering wake epochs and adjusted the S_on_, S_off_, TST (ie, S_off_ – S_on_), and WASO values accordingly. S_on_, S_off_, and REML are variables that are not provided directly by Fitbit; hence, we calculated them from the sleep staging information provided by Fitbit. All other variables were standard Fitbit variables. Adjustments only affected the Bland-Altman analyses. The results of the analyses without adjustment for the standard Fitbit variables can be found in Figure S1 and Table S1 in Multimedia Appendix 1. A sleep sensitivity setting is needed to be set for Fitbit’s sleep recordings, with options *sensitive* and *normal*. When set to *normal*, only major body movements, such as rolling over, will register as *wake*, whereas when set to *sensitive*, more subtle movements will additionally be registered as *wake*. We set the setting to *sensitive* throughout the data collection.

### Statistical Analyses

All analyses and data processing steps were performed in the programming language *R* (version 4.0.0; R Foundation for Statistical Computing) [33]. Fitbit intraday HR measures were used. For electrocardiogram R peak detection, the Pan-Tompkins algorithm [34] was used as implemented in the *rsleep* package (version 1.0.3) [35]. However, the algorithm could not distinguish sharp T waves from R peaks on various occasions. Thus, a modification of the algorithm had to be made. The signal can sometimes be inverted in the sign, and for this reason, we changed the signal to have positive R peaks (which was revealed by the mean of the detected peak values by the Pan-Tompkins algorithm). Sometimes, the peak can be slightly misaligned with the actual R peak maximum. Therefore, after running the Pan-Tompkins algorithm, the detected peak was aligned with the actual maximum ±200 ms around the detected peak. Furthermore, in cases where two peaks were observed within less than 360 ms, we checked if the subsequent peak was a mistakenly detected T wave or an actual R peak. This was done by examining the signal in a small window of ±28 ms around the detected and maximally aligned peak and taking its second derivative. T waves generally display slower changes in the tangents of the electrocardiogram signal as compared to faster tangent changes found in R peaks. The 60% quantile of the absolute value of the second derivative (QAVSD_60_) was then compared with a cutoff point specific to an individual participant derived from the density function of the QAVSD_60_ values from all the detected peaks. The cutoff point was defined as the first local minimum of the density within the hard limits of 35/256 µV/s^2^ and 120/256 µV/s^2^. If no local minimum was present, 35/256 µV/s^2^ was used instead. The density of QAVSD_60_ revealed a multimodal distribution of nearly no overlap between the T wave characteristic QAVSD_60_ values compared with those originating from R peaks. Erroneously detected T waves were omitted, thereby rescuing the affected segments of the electrocardiogram data sets for subsequent analyses. This small T wave check and alignment of the peak to the local maximum significantly improved the algorithm performance on visual inspection. From PSG IBIs, a transformation into bpm was made with 60 seconds divided by the IBI duration in seconds.

The internal clock times of the Fitbit and PSG systems were misaligned. This is a common problem in studies involving multiple measurement instruments, as they often do not share the same clock and thus require temporal alignment [36,37]. Hence, we estimated a time shift for each individual to ensure good time alignment. For this, linear interpolation was used to estimate values between two data points in either the PSG beat-per-beat data or the lower-resolution Fitbit data. We resampled both the Fitbit and PSG interpolated time series of a given night at 0.2-second intervals. The cross-correlation function was used to extract the lag with the maximal correlation between the time series.

Bland-Altman plots were constructed with the *blandr* package (version 0.5.1) [38] for all the sleep variables, two tailed *t* tests, and LoA defined as b (SD 1.96), where b denotes the bias and SD is the standard deviation of the bias. A variable is termed as *unbiased* if bias b is not significantly different from 0 from the corresponding *t* test. The differences in the Bland-Altman analyses were set to denote PSG *minus* Fitbit. Thus, a positive difference corresponds to an underestimation of Fitbit compared with PSG, and a negative difference corresponds to an overestimation. Concerning the repeated measurements of the 10%-trimmed HR average (HR_10_) and 10%-trimmed HR variance average (HRvar_10_) as measured at 1‑minute intervals, a linear mixed effects regression with the *nlme* package (version 3.1-147) was estimated [39]. The dependent variable was set to be the PSG-Fitbit value, and just a single intercept without a slope was considered the independent variable. For the random effect, a random intercept per subject was included. Owing to the consecutive 1-minute HR_10_ and HRvar_10_ measurements with potential time correlations, an autocorrelation structure of order one was added. The *t* tests and LoAs were estimated using a mixed model.

Epoch-by-epoch (EBE) analyses were performed through the following statistical measures:

Sensitivity = TP/P **(1)**

Specificity = TN/N **(2)**

Accuracy = (TP + TN)/(P + N) **(3)**

Matthews correlation coefficient (MCC) = (TP * TN - FP * FN)/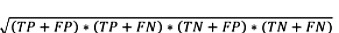

**(4)**

Positive predictive value (PPV) = TP/(TP + FP) **(5)**

Negative predictive value (NPV) = TN/(TN + FN) **(6)**

In these equations, TP represents true positives (number of Fitbit epochs that share a given PSG stage), TN represents true negatives (the number of Fitbit epochs that are not in a given stage and where the according PSG epoch is also not labeled as that stage), FP represents false positives (number of Fitbit epochs that do not share a given PSG stage), and FN represents false negatives (number of Fitbit epochs that did detect a given stage, whereas PSG did not detect it). Sensitivity measures the proportion of epochs of a given PSG-derived sleep state that was correctly identified by Fitbit (eg, for REM sleep, it is the percentage of Fitbit *REM* sleep stages among all PSG REM sleep stages). Specificity, however, describes the percentage of Fitbit correctly identifying the nonoccurrence of a given sleep state. Accuracy is a combined measure of the true discoveries and true negatives of Fitbit divided by all positives and negatives in the PSG sample. MCC is more informative than the measure accuracy, because it considers all true positive, true negative, false positive, and false negative. This can be interpreted as a correlation coefficient, that is, the more positive, the better Fitbit predicts the PSG epochs, such that 0 would be random guessing, and negative values indicate disagreement. PPV, often called precision, describes the proportion of Fitbit correctly identifying a given stage among the number of times Fitbit assigned that stage, and NPV describes the equivalent for correctly identifying an epoch that is not a given stage. In our sample, the epoch length was defined as 20 seconds, but Fitbit’s algorithm has an epoch length of 30 seconds. Thus, a direct EBE analysis was not possible. Therefore, we looked at all PSG-derived epochs and compared them with the dominating Fitbit stage (>50%) in the same interval. In cases where one PSG epoch contained two different Fitbit stages of equal length, we chose the first stage.

## Results

### Demographic Characteristics of the Study Sample

The demographics of the 59 individuals studied as well as their mean PSG- and Fitbit-derived sleep and HR measures are summarized in Tables 1 and 2. The mean values on the PSQI and PSS-10 indicated slightly impaired subjective sleep quality and a slightly elevated perceived stress level [27]; however, no diagnostic criteria for possible posttraumatic stress disorder have been met [28].

**Table 1:** Demographics of study sample (N=59).

	Value
Female, n (%)	33 (56)
Police, n (%)	15 (25)
Age (years), mean (SD)	33.5 (8.1)
BMI, mean (SD)	23.9 (2.9)
PSQI^a^, mean (SD)	5.8 (2.7)
PCL-5^b^, mean (SD)	6.2 (7.9)
PSS-10^c^, mean (SD)	12.2 (4.9)
rMEQ^d^, mean (SD)	14.4 (3.5)

^a^PSQI: Pittsburgh Sleep Quality Index.

^b^PCL-5: Posttraumatic Stress Disorder Checklist for Diagnostic and Statistical Manual of Mental Disorders Fifth Edition.

^c^PSS-10: Perceived Stress Scale 10.

^d^rMEQ: Horne-Östberg Morningness-Eveningness Questionnaire-A Reduced Scale.

**Table 2:** Sleep and heart rate variables (N=59).

	Value, mean (SD)	
	Polysomnography	Fitbit
N1_so_^a^ (clock time)	23.4 (0.9)	23.4 (2.4)
TST^b^ (hours)	8.0 (1.7)	7.8 (2.6)
REM_d_^c^ (hours)	1.7 (0.8)	1.7 (0.7)
light_d_^d^ (hours)	4.2 (1.1)	4.4 (1.3)
deep_d_^e^ (hours)	1.5 (0.6)	1.3 (0.5)
WASO^f^ (hours)	0.4 (0.5)	1.0 (1.1)
REML^g^ (minutes)	76.3 (30.6)	103.9 (59.7)
REM^h^ in the first cycle (%)	11.6 (8.1)	15 (8.7)
HR_10_^i^ REM (bpm^j^)	60.9 (9.1)	59.9 (8.2)
HR_10_ N1^k^ (bpm)	61.8 (9.2)	59.2 (7.5)
HR_10_ N2^l^ (bpm)	56.6 (7.7)	55.7 (7.0)
HR_10_ N3^m^ (bpm)	58.8 (8.8)	57.2 (7.2)
HRvar_10_^n^ REM (bpm)	28.1 (90.8)	6.4 (16.1)
HRvar_10_ N1 (bpm)	48.7 (110.1)	6.8 (16.7)
HRvar_10_ N2 (bpm)	22.0 (76.7)	4.7 (24.3)
HRvar_10_ N3 (bpm)	25.4 (111)	2.9 (12.9)

^a^N1_so_: sleep onset with non–rapid eye movement (NREM) sleep stages 1 criteria.

^b^TST: total sleep time.

^c^REM_d_: rapid eye movement sleep duration.

^d^light_d_: light sleep or NREM sleep stages 1+NREM sleep stages 2 duration, respectively.

^e^deep_d_: deep sleep or NREM sleep stages 3 duration, respectively.

^f^WASO: wakefulness after sleep onset.

^g^REML: rapid eye movement sleep latency.

^h^REM: rapid eye movement.

^i^HR_10_: 10%-trimmed heart rate average.

^j^bpm: beats per minute.

^k^N1: NREM sleep stages 1.

^l^N2: NREM sleep stages 2.

^m^N3: NREM sleep stages 3.

^n^HRvar_10_: 10%-trimmed heart rate variability.

### Time Alignment

**Figure 1:**
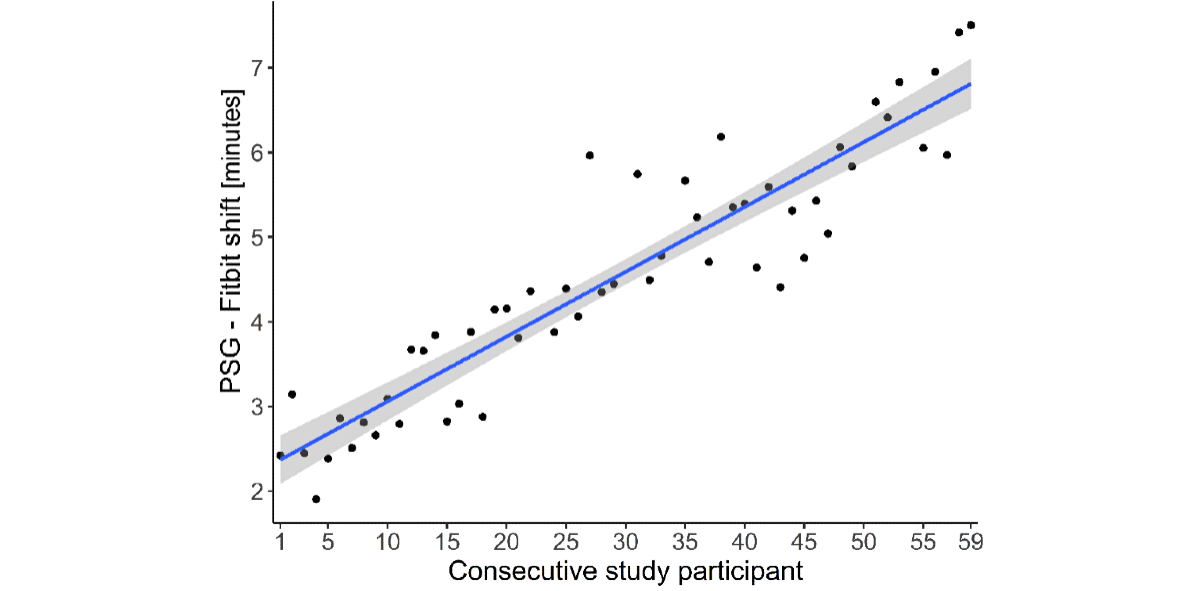
The consecutive study participant numbers (higher numbers indicate chronologically later entry into the study) from the entire study sample are shown on the x-axis; the data-driven timeshift between polysomnography and Fitbit is shown on the y-axis. There was a significant linear relationship between the identifier and the shift (*P*<.001; adjusted R^2^=0.85). Thus, the times drifted apart as the study went on, with a minimum time misalignment of 1.9 minutes and a maximum of 7.5 minutes. PSG: polysomnography.

Accurate temporal synchronization between the PSG system and the wearable Fitbit device often poses a methodological challenge in validation studies [4]. This was also the case in this study. When scrutinizing our data, we noticed that the time discrepancies between the PSG system’s and the Fitbit app’s clocks increased as the study progressed. In other words, the later the participant entered the study, the higher the time difference between PSG and Fitbit recordings. This relationship can be seen in Figure 1 as a linear association between the individual participant identifier number and the estimated time shift between the two measurement instruments.

**Figure 2:**
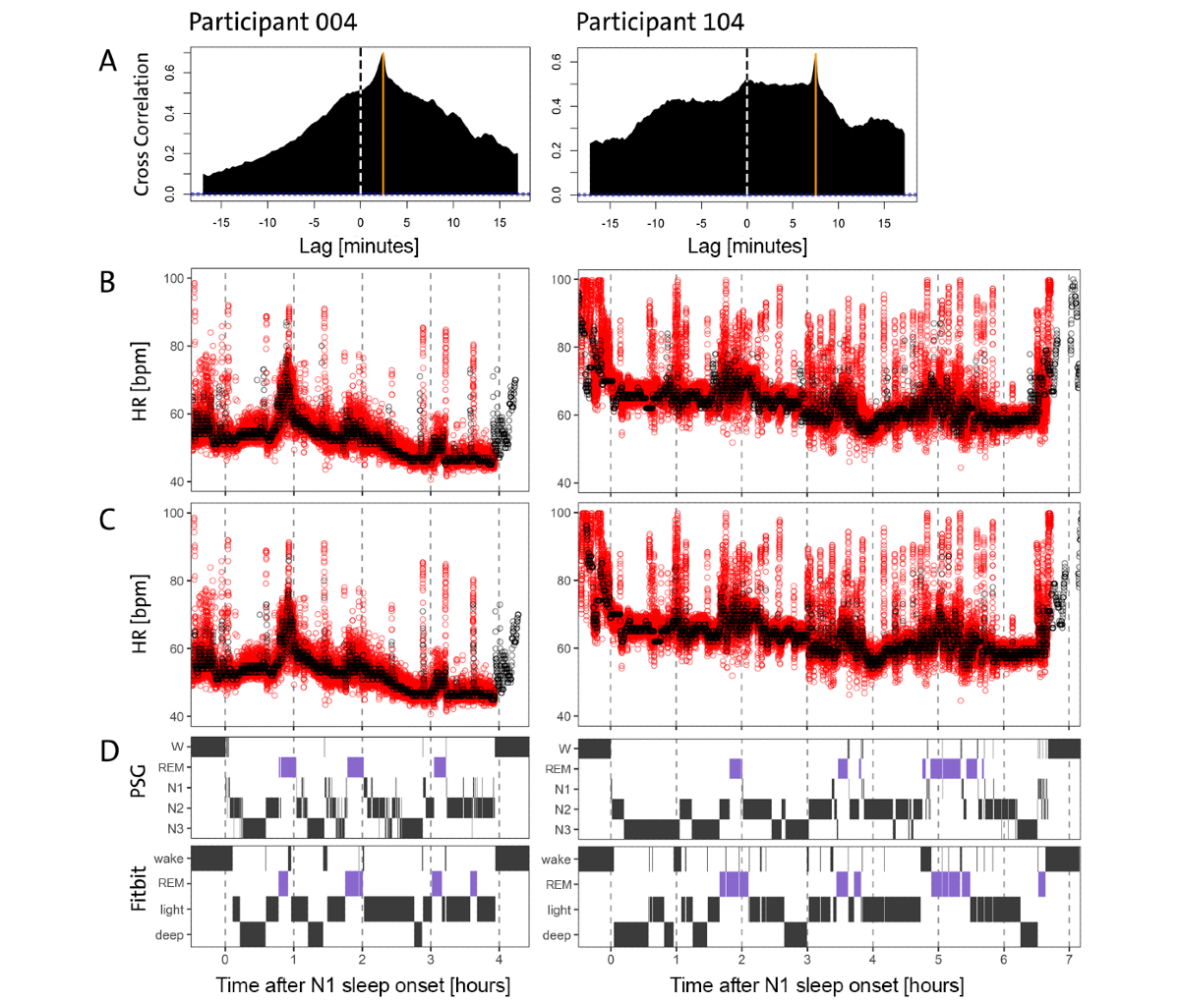
Data on the validation night of the first participant in the study with identifying number 004 (left column) and the last participant in the study with number 104 (right column) are shown. Row A displays the cross-correlation function, which displays a large visible maximum at the orange vertical line representing the best alignment between the two devices (PSG and Fitbit). The dashed vertical reference line shows a lag of 0 minutes. Rows B-D share the same x-axis, which denotes hours after PSG-derived sleep onset with criteria. For each hour in the recording, a vertical dashed gray line was added. Row B shows the HR in bpm derived from PSG (red) and Fitbit (black) that were seen before any time alignment was applied, whereas row C presents the HR data after the data-driven shift from panel A was applied. The time-aligned time series visually shows good agreement after correcting for the time difference. Fitbit shows reduced variability in the signal but fairly good average correspondence. In panel D, the top row shows PSG-derived hypnograms for both participants, whereas in the bottom row, the Fitbit-derived hypnograms are displayed. All hypnograms have been time-corrected according to panel A. The overall sleep structure is captured reasonably well by Fitbit, but Fitbit detects more wake and REM episodes compared with PSG, and the distinction of light (N1+N2) and deep (N3) sleep often seems to be particularly challenging for Fitbit. bpm: beats per minute; HR: heart rate; PSG: polysomnography; REM: rapid eye movement; W: wake.

**Figure 3:**
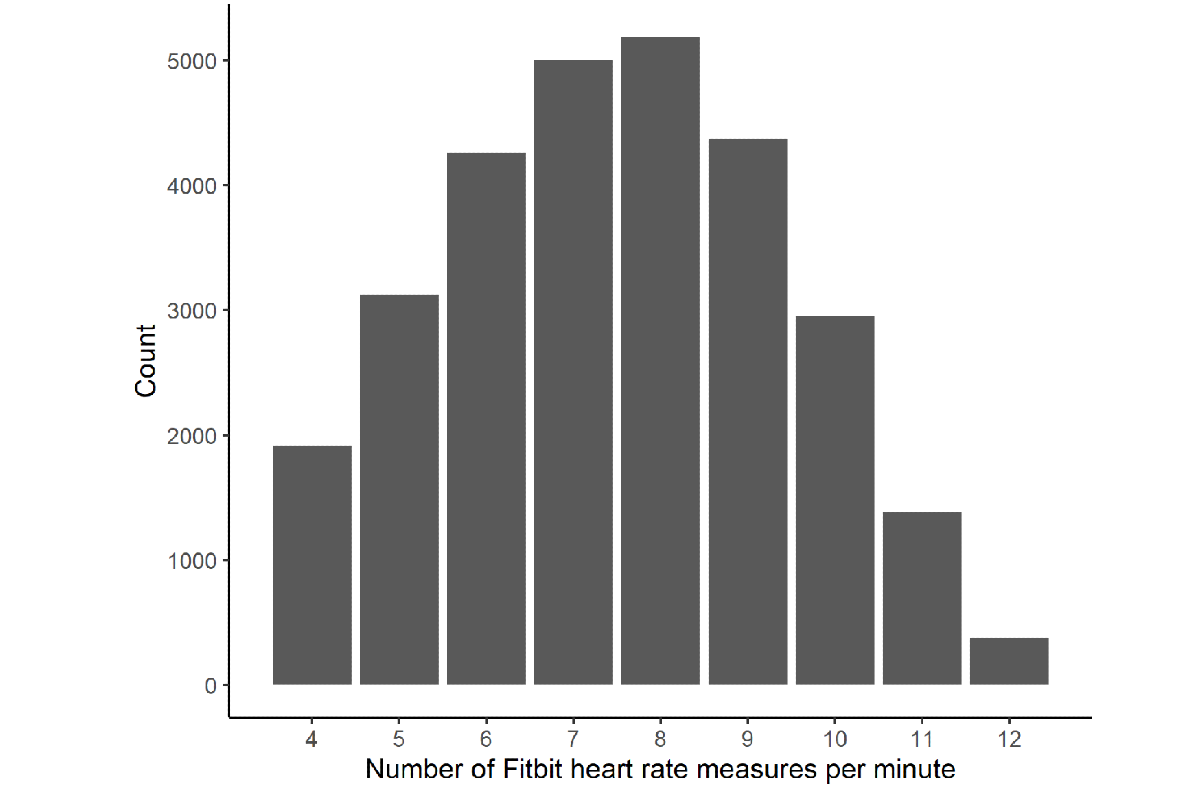
The available data of all nights (n=59) were extracted and counted for the number of heart rate measures contained. A total of roughly 28,320 minutes (corresponding to 59 study participants who, on average, spent 8×60 minutes asleep) were expected. In fact, 28,601 individual minutes of data were recorded; this figure displays the distribution of all heart rate measures, yielding an average of 7.48 measures per minute. Count data for >12 measures per minute and <4 measures per minute are not displayed because their occurrences were so small that they are not visible on the plot.

To align the time series, we computed the cross-correlation function for each participant and corrected the time shift by the emergent maximum. Our time alignment efforts produced good correspondence in our data between the two instruments, as evident in the simultaneous occurrences of HR bursts in the two time series (Figure 2). Nevertheless, the variability and amplitude of the Fitbit curve were reduced compared with PSG because only between 4 and 12 measurements per minute were made available by Fitbit. The analysis of the entire Fitbit sample revealed that an average of 7.48 HR counts per minute was available (Figure 3). In contrast, PSG HR data were sampled at a frequency of 1/256 Hz.

### Distribution of Sleep Stage Durations

**Figure 4:**
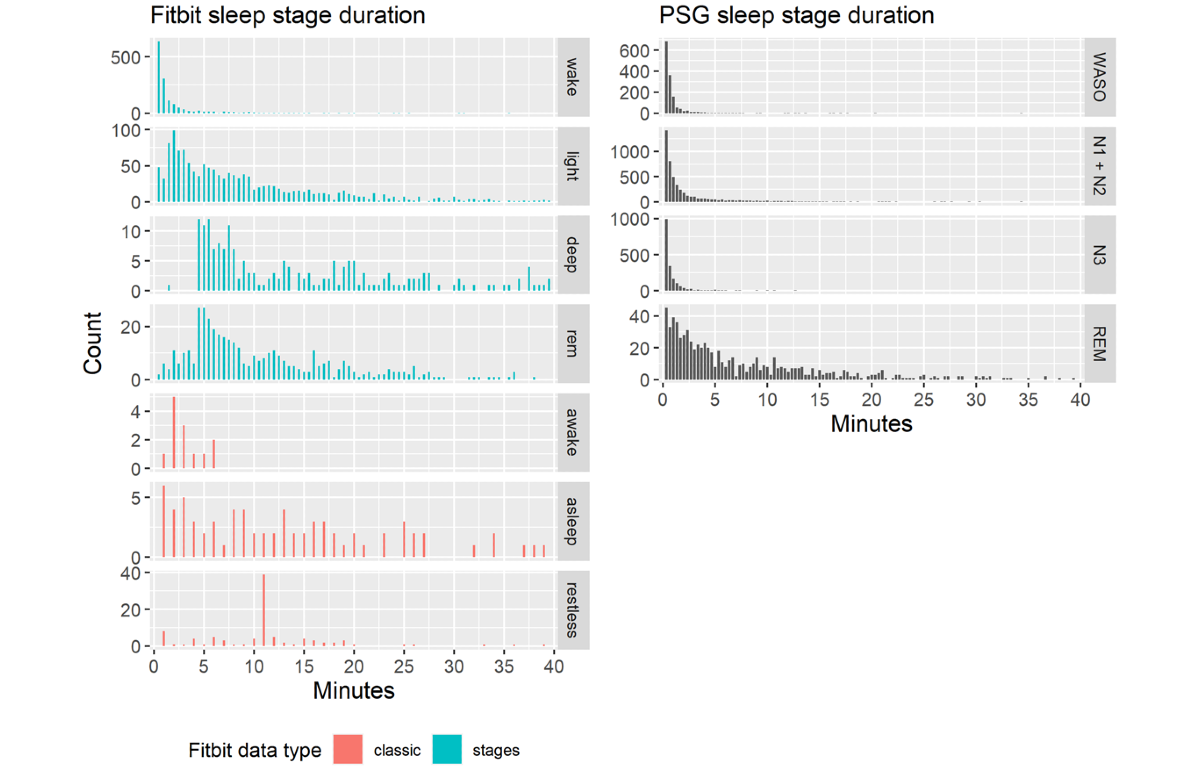
The distribution of sleep stage durations for Fitbit (left panel) and PSG (right panel). Both were computed on the sample of the nights used for validation. Here, the plot has been cut off at 40 minutes for visual purposes; the tails continue to decrease as one would expect. The Fitbit sleep staging data types "classic" (red) and "stages" (blue) show large deviations compared with PSG sleep stages (black). Of note, deep and REM sleep show nonbiological discontinuity at around 4.5 minutes, and all Fitbit stages have larger tails. The stage "restless" has a peak at 11 minutes with unknown meaning. PSG: polysomnography. REM: rapid eye movement; WASO: wakefulness after sleep onset.

Next, we compared the distribution of sleep stage durations between the Fitbit and PSG data (Figure 4). Duration was defined as the duration of consecutive epochs with the same sleep stage until interrupted by any other stage, independent of its duration. We observed that Fitbit uses 30-second intervals to classify the *stages* data, whereas the *classic* data are presented with less time-resolved, 1-minute resolutions. With respect to wake episodes, the Fitbit data resembled the PSG distribution, with mostly short uninterrupted wake episodes and much rarer longer episodes. The *awake* category in the *classic* datatype had higher tails, possibly owing to having a resolution of 1 minute instead of 30 seconds, thus potentially missing certain stage changes that occur faster. On the basis of the inspection of the data distributions, we assumed that Fitbit’s *light* sleep stage in the *stages* datatype might capture PSG-defined N1+N2 sleep stages, whereas *deep* sleep might capture PSG-defined N3 sleep. However, these assumptions need to be treated with caution because no information is provided by Fitbit. In general, *light* and *deep* sleep showed longer tails than the PSG-defined non–rapid eye movement (NREM) sleep stages, possibly owing to different temporal resolutions or slower changes in HR and HR variability compared with the more sudden changes in brain states. Furthermore, the *deep* sleep distribution showed a pronounced discontinuity at around 4 minutes and 30 seconds, which could also be observed in the Fitbit *REM* sleep stage duration. The distributions of *light*, *deep*, and *REM* sleep showed discrepancies to the PSG-derived durations, indicating that the algorithm does not fully reflect PSG-derived data and may miss brief stage changes and stage interruptions. Furthermore, the *restless* stage in the *classic* datatype is unknown. This stage displayed a peak at approximately 11 minutes, with an unknown origin.

### Bland-Altman Analyses of Sleep Variables

**Figure 5:**
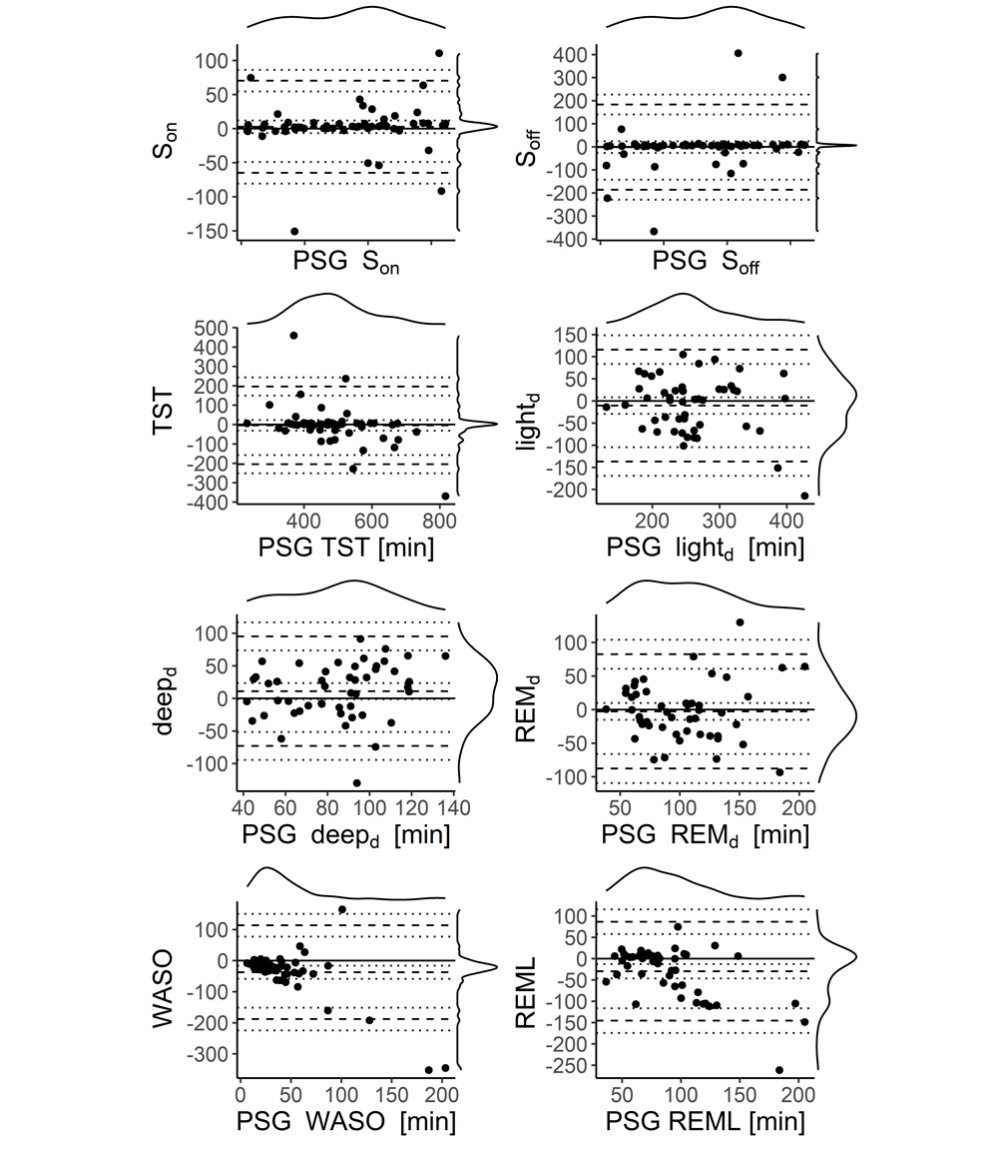
Bland-Altman plots for various sleep variables are shown with sleep onset defined as the first occurrence of N1. The dashed lines denote lower limits of agreement, bias, and upper limits of agreement. The dotted lines are the respective 95% CI of limits of agreement. On the top and right of each panel, the marginal densities are plotted. The x-axis displays the PSG variables, and the y-axis denotes the differences between the two devices (PSG-Fitbit). N1-derived sleep onset is unbiased. Sleep offset, total sleep time, light sleep or N1+N2 sleep duration, deep sleep or N3 sleep duration, and REMd do not have significant bias. WASO and REML display a significant deviation of the difference between the devices from 0. deep_d_: deep sleep duration; light_d_: light sleep duration; PSG: polysomnography; REM_d_: rapid eye movement sleep duration; REML: rapid eye movement sleep latency; S_off_: sleep offset; S_on_: sleep onset; TST: total sleep time; WASO: wake after sleep onset.

We split our validation into two analyses, one with the PSG-determined first occurrence of N1 sleep as the criterion for S_on_ (N1 S_on_ [N1_on_]) and the other with the first occurrence of N2 sleep as the criterion for S_on_ (N2 S_on_ [N2_on_]). This was done because it is unknown how Fitbit estimates S_on_. In Figure 5, we plotted the variables computed with N1_on_, and Table 3 provides the associated statistics. The N2_on_ analyses revealed systematically higher biases. These data are presented in Figure S2 and Table S2 in Multimedia Appendix 1. S_on_, defined as N1_on_, was unbiased (–1.6 minutes; *P*=.73). S_off_, TST, REM sleep duration (REM_d_), the duration of Fitbit’s *light* sleep duration (light_d_) in minutes (as recorded by the Fitbit; interpreted as N1+N2), and the *deep* sleep duration (deep_d_) in minutes (as recorded by the Fitbit) did not display significant bias. Nevertheless, deep_d_ showed a trend toward a bias of 11.2 minutes with N1_on_ (*P*=.08), likely pointing to a slight underestimation with Fitbit of N3 sleep. REML and WASO both exhibited a significant overestimation with Fitbit—REML was overestimated by 29.4 minutes and WASO by 37.1 minutes (*P*_all_<.001). Although the marginal densities of the differences for S_on_, S_off_, and TST were quite narrow, indicating a good estimator in general, some occasional sleep episodes disagreed strongly between the Fitbit and PSG instruments, as reflected in the large LoA (Table 3). The marginal distributions of REM_d_, light_d_, and deep_d_ showed higher variance, even if outliers were neglected. This observation may indicate that the estimation of stages of sleep is challenging for Fitbit’s algorithm and a source of variability, although being unbiased. The data on the standard Fitbit variables without the bordering wake epoch adjustment revealed very similar results, however, with slightly larger biases for TST and WASO, as shown in Figure S1 and Table S1 in Multimedia Appendix 1.

**Table 3:** Bland-Altman statistics^a^.

^Variable^			PSG^b^-Fitbit		Lower LoA^c^		Upper LoA		*P* value
S_on_^d^ (minutes)			–1.6		–68.8		65.6		.73
S_off_^e^ (minutes)			–5.6		–189.3		178.2		.66
TST^f^ (minutes)			–4.0		–204.3		196.3		.77
REM_d_^g^ (minutes)			–2.7		–87.8		82.4		.67
light_d_^h^ (minutes)			–10.4		–136.8		116.0		.27
deep_d_^i^ (minutes)			11.2		–72.9		95.2		.08
WASO^j^ (minutes)			–37.1		188.1		113.8		.001
REML^k^ (minutes)			–29.4		–145.4		86.6		.001
**HR_10_^l^ (bpm^m^)**									
	Overall	0.9		–6.9		8.6		<.001	
	WASO	1.9		–5.4		9.2		.03	
	N1^n^	1.2		–8.9		11.3		.14	
	N2^o^	0.6		–4.7		6.0		.001	
	N3^p^	0.6		–6.4		7.6		.008	
	REM^q^	0.7		–4.7		6.0		<.001	

^a^Statistics accompanying the Bland-Altman plots (Figure 5). Sleep onset and rapid eye movement (REM) sleep latency were calculated using the non–rapid eye movement (NREM) sleep stages 1 sleep onset criteria. The average 10%-trimmed heart rate and 10%-trimmed heart rate variance values in various sleep states are presented in the columns below the sleep variables. The average difference between polysomnography and Fitbit measures bias can be found in the first column. The lower and upper limits of agreement describe 1.96 times the SD around the bias and can be found in the subsequent columns. In the last column, the *P* values for the paired *t* test are reported; we tested whether the bias was significantly different from 0.

^b^PSG: polysomnography.

^c^LoA: limit of agreement.

^d^S_on_: sleep onset.

^e^S_off_: sleep offset.

^f^TST: total sleep time.

^g^REM_d_: REM sleep duration.

^h^light_d_: light sleep duration.

^i^deep_d_: deep sleep duration.

^j^WASO: wakefulness after sleep onset.

^k^REML: REM sleep latency.

^l^HR_10_: 10%-trimmed heart rate average.

^m^bpm: beats per minute.

^n^N1: NREM stage 1 sleep.

^o^N2: NREM stage 2 sleep.

^p^N3: NREM stage 3 sleep.

^q^REM: rapid eye movement.

### Bland-Altman Analyses of HR Variables

**Figure 6:**
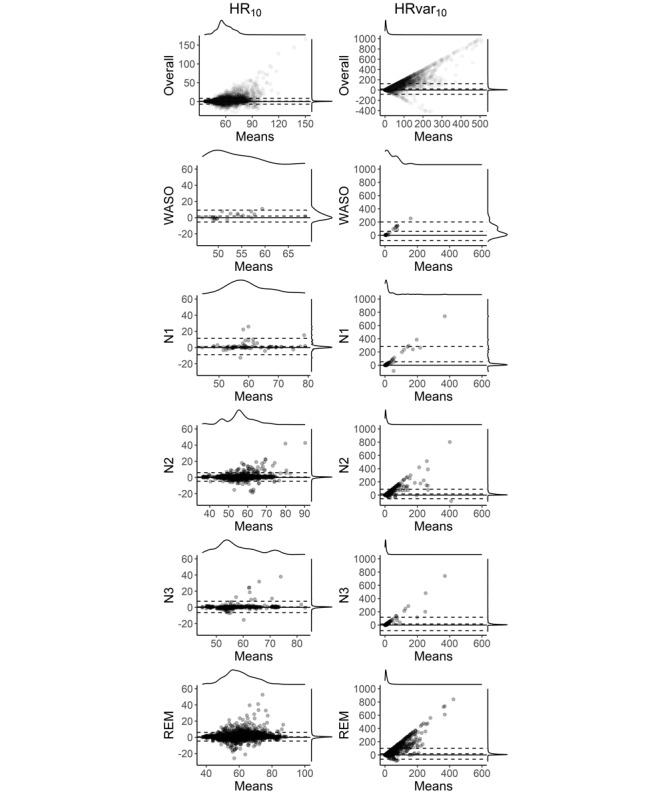
Bland-Altman plots for heart rate–derived variables. The dashed lines denote lower limits of agreement, bias, and upper limits of agreement for a mixed model dealing with the repeated measures. On the top and right of each panel are the marginal densities. The x-axis displays the means of both devices (ie, [polysomnography + Fitbit]/2), and the y-axis denotes the differences between the two devices (polysomnography-Fitbit). Overall average 10%-trimmed heart rate and 10%-trimmed heart rate variance values are calculated for 1-minute intervals between 30 minutes before sleep onset with N1 criteria and 30 minutes after sleep offset. All other variables are calculated between sleep onset and sleep offset, only extracting the designated variable, in 1-minute intervals. HR_10_: 10%-trimmed heart rate average; HRvar_10_: 10%-trimmed heart rate variance average; REM: rapid eye movement; WASO: wake after sleep onset.

The Bland-Altman plots for the HR variables are shown in Figure 6. When computing the interval between 30 minutes before N1_on_ until S_off_ without considering the different wakefulness and sleep states, HR_10_ and HRvar_10_ measures both appeared biased. More specifically, Fitbit underestimated HR_10_ overall by 0.9 bpm and displayed LoA of –6.9 and 8.6 bpm (Table 3). This underestimation was rather small, with a relatively narrow marginal distribution of the differences. When focusing on 1-minute HR_10_ values restricted to the time interval between S_on_ and S_off_ and dividing among the PSG-derived states N1, N2, N3, REM sleep, and wake, HR_10_ displayed a higher bias in the wake (1.9 bpm; *P*=.03) and N1 (1.2 bpm; *P*=.14) stages compared with the sleep stages N2 (0.6 bpm; *P*=.001), N3 (0.6 bpm; *P*=.008), and REM sleep (0.7 bpm; *P*<.001).

When analyzing overall HR variance, Fitbit strongly underestimated HRvar_10_ with a bias of 20.3 bpm (*P*<.001), which was associated with higher LoA –82.1 and 122.7. When HRvar_10_ was divided among the different sleep stages, we observed behavior similar to HR_10_, such that HRvar_10_ wake and N1 had a higher bias (60.2 and 51.1 bpm) than N2, N3, and REM sleep (17.6, 16.3, and 18.5 bpm), all with low *P* values and considerably large LoA.

### EBE Analysis

The EBE analysis results are displayed in Table 4. The EBE comparison between Fitbit and PSG revealed that Fitbit displayed better specificity (WASO: 0.898; light sleep as N1+N2: 0.574; deep sleep as N3: 0.92; REM sleep: 0.889) than sensitivity (WASO: 0.428; light sleep as N1+N2: 0.534; deep sleep as N3: 0.279; REM sleep: 0.548). The sensitivity for REM sleep was worse during the initial 120 minutes of sleep (0.432) when compared with REM episodes beginning 120 minutes or more after S_on_ (0.57). In contrast, for specificity, this relationship was reversed (REM<120 minutes: 0.963; REM>120 minutes: 0.864). Accuracy was best for WASO (0.898) and REM sleep (0.880) and worse for deep sleep N3 (0.776) and light sleep N1+N2 (0.553). A similar relationship was reflected in the MCC, ranging from weak to moderate correlation (REM sleep: 0.339; WASO: 0.329; deep sleep as N3: 0.25; light sleep as N1+N2: 0.108). The MCC measure is preferable to accuracy as it only leads to higher scores if the prediction is simultaneously accurate in all confusion matrix categories (true positive, false positive, true negative, and false negative) [40]. PPV, the probability that an episode with a given Fitbit stage will also have the same PSG stage, was generally lower (WASO: 0.438; light sleep as N1+N2: 0.592; deep sleep as N3: 0.501; REM sleep 0.306) compared with NPV, the probability that an episode that does not have a certain Fitbit stage will also not have that PSG stage (WASO: 0.894; light sleep as N1+N2: 0.516; deep sleep as N3: 0.815; REM sleep: 0.956).

**Table 4:** Epoch-by-epoch analysis^a^.

State	Sensitivity	Specificity	Accuracy	MCC^b^	PPV^c^	NPV^d^
WASO^e^	0.428	0.898	0.824	0.329	0.438	0.894
Light sleep	0.534	0.574	0.553	0.108	0.592	0.516
Deep sleep	0.279	0.920	0.776	0.250	0.501	0.815
REM^f^ sleep	0.548	0.889	0.861	0.339	0.306	0.956
REM sleep <120 minute	0.432	0.963	0.934	0.383	0.403	0.967
REM sleep >120 minute	0.570	0.864	0.837	0.329	0.296	0.953

^a^Epoch-by-epoch comparison of Fitbit and polysomnography stages.Each stage—wakefulness after sleep onset, light sleep (non–rapid eye movement [REM] stage 1 [N1] sleep+NREM stage 2 sleep), deep sleep (NREM stage 3 sleep), and REM sleep—was analyzed. REM sleep was divided into analyses with REM sleep episodes occurring during the first 120 minutes after sleep onset with N1 sleep criteria (N1 sleep onset) and REM sleep episodes occurring later than 120 minutes after N1 sleep onset. Various performance measures were used, including sensitivity, specificity, accuracy, the Matthews correlation coefficient, the positive predictive value, and the negative predictive value. More information on these measures can be found in the *Methods* section. Fitbit showed mostly good specificity but poor sensitivity. The accuracy was relatively high except for the light sleep stage. The Matthews correlation coefficient displayed a moderately positive relationship, with light and deep sleep being considerably less good. The negative predictive value was usually higher than the positive predictive value.

^b^MCC: Matthews correlation coefficient.

^c^PPV: positive predictive value.

^d^NPV: negative predictive value.

^e^WASO: wakefulness after sleep onset.

^f^REM: rapid eye movement.

## Discussion

### Principal Findings

We evaluated the performance of the multisensor wearable Fitbit Charge 2 against PSG of the sleep macrostructure and HR in a sample of first responder shift workers under naturalistic conditions. We observed that S_on_, S_off_, TST, REM_d_, N1+N2 sleep duration, and N3 sleep duration showed unbiased estimates but nonnegligible LoA. Fitbit overestimated REML by –29.4 minutes, possibly because the proprietary algorithm failed to detect very short first REM sleep episodes. This hypothesis is supported by the right shift in the maximum duration of stages and larger tails (Figure 4) and a cluster of REML data points occurring at approximately –100 minutes (Figure 5), indicating that Fitbit cannot capture short-lasting stage durations well. Not only REML but also other sleep variables often exhibited a wide LoA. In addition, despite performing a careful, data-driven time alignment between the Fitbit and PSG time series, which differed from 1.9 minutes to 7.5 minutes depending on the participants’ entrance into the study, Fitbit overestimated WASO by as much as 37.1 minutes. We concluded that the unbiased sleep variables allow average estimations of important sleep quality characteristics in ecological conditions. However, the wide LoA in most variables and the large biases in REML and WASO limited the meaningfulness of quantifying individual sleep episodes. These findings highlight the considerable challenges still present when relying on consumer-grade technology to address clinical and research questions.

One of our most striking and novel findings is that the distribution of all sleep episode durations differs between the Fitbit Charge 2 and PSG. Fitbit’s sleep staging algorithm probably treats *REM* and *deep* sleep states of less than 4.5 minutes differently than sleep stages exceeding this duration. This introduces a nonbiological discontinuity, indicating the potential limitations of the tracker’s staging algorithm. Furthermore, it is not clear what PSG measurement corresponds to the Fitbit stage *restless*, which renders meaningful comparisons impossible. Our findings in the sleep episode duration distribution are consistent with recent work [41], which also revealed an underestimation of sleep stage transition dynamics.

The S_on_ measures from Fitbit were unbiased concerning the N1_on_ criteria, whereas there was a higher but nonsignificant underestimation for N2_on_. Thus, it is likely that Fitbit’s definition of S_on_ time roughly corresponds to PSG-derived N1_on_. S_on_ criteria should be reported in future validation studies because whatever criterion one selects (eg, N1_on_, N2_on,_ or alternatively any stage of sleep) will impact many sleep variables, such as TST, REML, and WASO, whose operational definition and calculation depend upon the criterion of S_on_. This may be one of the reasons for discrepancies reported in the validation literature. A peculiarity of the staging information provided by Fitbit is that the first stage after the S_on_ time and the last stage before S_off_ time is sometimes staged as *awake* or *wake*. We manually adjusted the S_on_ and S_off_ times to be delineated by the first and last occurring stages of sleep rather than including stages of wake at the border of sleep. In a large Fitbit data set collected in 89 individuals for 1 month capturing roughly 3000 sleep episodes [42], 69.8% of all sleep episodes in the first stage after S_on_ and in 50% of all cases, the last stage before S_off_ was not coded as a sleep stage. In other words, an appreciable proportion of Fitbit sleep episodes are *bookended* by a stage of wake. This is an inconspicuous but important caveat. Our adjustment of these data could be a reason why we found N1_on_, S_off_, and TST to be unbiased when comparing Fitbit data with PSG data, whereas Liang et al [16], de Zambotti et al [43], and Morena-Pino et al [23] found TST biases in different directions. More specifically, a previous study [43] found unbiasedness; another reported an overestimation of WASO [16], whereas Moreno-Pino [23] found an underestimation of WASO when validating Fitbit Charge 2 against PSG. The study by Liang et al [16] with a WASO bias of 24.5 minutes is closest to our results of 37.1 minutes.

Overall, EBE analyses revealed better specificity than sensitivity for all sleep states. This might have been expected. For example, there are much fewer *deep* sleep epochs than epochs labeled as any other sleep stage, which is why a single misclassification carries more weight for sensitivity than specificity. We found *light* sleep to have 0.55 accuracy, whereas de Zambotti et al [43] found an accuracy of 0.81. However, the same study found an accuracy of 0.49 for *deep* sleep, whereas we found a higher respective value of 0.78. Furthermore, REM sleep showed an accuracy of 0.86, similar to that of 0.74 found by de Zambotti et al [43]. A recent systematic review (Haghayegh [14] on various Fitbit devices including Alta, Alta HR, Charge 2, Charge HR, Classic, Flex, One, Surge, Ultra and Versa models) found accuracy values in the range of 0.69-0.81 for *light* sleep, 0.36-0.89 for *deep* sleep, and 0.62-0.89 for REM sleep. Thus, our results for *light* sleep are slightly lower than the range suggested previously, whereas, for *deep* sleep and REM sleep, the accuracy in our study was in the upper range reported. The MCC value, which can be interpreted as a usual correlation coefficient, ranged from 0.11 in *light* sleep to 0.34 in REM sleep. These numbers indicate low to medium strength of correlation, pointing toward room for improvement in the estimation of sleep stages by Fitbit.

The information Fitbit provides on the sleep sensitivity setting, with options *sensitive* and *normal*, may have an influence on the amount of stages that are scored as wake [44]. We set the setting to *sensitive* when data were collected, which might have led to an overestimation of WASO, as seen in Figure 2. However, Fitbit states that this setting has no impact on devices utilizing HR to track sleep [45]. Consistent with our results, REM_d_ was also found to be unbiased by [3]. In addition, we found light_d_ and deep_d_ to be unbiased. As the algorithm is not open source, we do not know with certainty whether our study was running on an updated version of the algorithm compared with other validation studies. This limitation makes it difficult to compare the validation study outcomes of consumer fitness trackers in general [4] and could contribute to the discrepancies with the previous literature. Another reason might stem from the different populations sampled or recording conditions. For example, the algorithm might be better suited to assess sleep in healthy individuals than in patients or shift workers or may perform better in a sleep laboratory than in a naturalistic environment. The discrepancies among studies underscore the necessity to define standardized procedures to test consumer sleep technology to benefit from their potential to collect large-scale sleep data in ecological conditions [21,22,26].

Regarding the HR data, Fitbit slightly underestimated overall HR_10_ by 0.9 bpm with a limited capability to capture sudden HR changes. This underestimation was smaller in N2, N3, and REM sleep stages (0.6, 0.6, and 0.7 bpm, respectively) compared with N1 sleep and wake (1.2 and 1.9 bpm), thus indicating a sleep stage–specific bias. The bias was low and probably not biologically relevant. The low *P* values of biases in differences in the HR measures between the devices arise from the repeated measure design as a vast number of 1-minute values during the whole night for each subject was calculated, thereby increasing the statistical power to detect small biases as significant. The evident HR bias of 0.9 bpm is strikingly similar to the HR bias of 0.88 bpm found in de Zambotti et al [25] in the related Fitbit Charge HR device. As mentioned in the report by Haghayegh et al [13], Fitbit Charge HR and Fitbit Charge 2 share the same hardware and software, thus making a comparison feasible, software updates notwithstanding. We found a stage-dependent bias with lower underestimation in deeper sleep stages sharing lower HR on average and a larger underestimation in wake state and a more transitory sleep stage N1, which share higher HR values on average, a finding compatible with the HR-dependent bias reported by Haghayegh et al [13]. For an HR during sleep of <50 bpm, these authors found an overestimation of 0.51 bpm, and for an HR during sleep >80 bpm, an underestimation of 0.63 bpm. These values are comparable with our findings. On the other hand, Benedetto et al [24] found an HR underestimation of 5.9 bpm during wake state. We also found a larger underestimation during wake episodes of 1.2 bpm, but not as high as 5.9 bpm. In the study by Benedetto et al [24], no time alignment between the two instruments was reported. The method of capturing HR via video recording of live values displayed on the Fitbit app was innovative but could be a source of error. Hence, the results could potentially be influenced by a timing misalignment between the instruments and data collection methods.

Fitbit HR variance was reduced owing to the inaccessibility of raw data and showed higher LoA than the LoA for HR. The differences between the assessments are not surprising, as Fitbit only provided 7.4 measurements per minute on average (Figure 3). This is probably owing to their algorithm providing some averaged values in preferably 5 seconds, 10 seconds, and 15 seconds measurement intervals (but other interval lengths, eg, 2-second or 7-second intervals, can also be found in the data). For comparison, a PSG-derived HR value can be computed for each IBI. Thus, receiving preprocessed data from Fitbit instead of raw data naturally leads to a considerably higher variance in PSG recordings. Moreover, all HR values from Fitbit are integers, whereas the values from the PSG are real values. This difference in the nature of the values (rounded to integers) additionally leads to slightly different behaviors of the HR_10_ and HRvar_10_ measures. The Fitbit photoplethysmography would be able to capture brief bursts in HR, as evidenced by a study on exercising awake individuals [24]. Data with approximately 1-second time resolutions are only made available in the device’s *exercise* mode, which prevents sleep tracking. Nevertheless, Fitbit may still be able to detect variability changes for longer periods during sleep with a reasonable degree of accuracy even without providing users with high resolution or raw HR data (as seen in Table 1, where the ordering of the variance per sleep stage remains nearly intact between Fitbit and PSG).

### Limitations

The missing information regarding an objective marker of *lights out* is a limitation of our study, which prevented us from estimating sleep latency. In addition, the number of measurements per minute provided by Fitbit varied, potentially owing to variable signal quality and other internal decision-making processes in Fitbit’s proprietary data preprocessing algorithms. Updates to software or firmware could have occurred without notice, harboring a great potential to confound research or clinical undertaking, particularly in longitudinal scenarios. Individual sleep episodes can vary appreciably even within an individual, and caution should be exercised when interpreting results from a Fitbit device. Not being able to blind participants to their own sleep data after collection could influence their behavior in subsequent sleep episodes. This concern is particularly pressing when clinical or otherwise vulnerable populations are involved, and device output is interpreted, which may impact treatment options or health outcomes. For this reason, it is crucial that these devices be validated in more clinically diverse populations.

### Conclusions

In a study conducted at home in a relatively large sample validating Fitbit Charge 2 against PSG, compared with most previous validation studies (n=15 [24]; n=25 [16]; n=35 [43]; n=35 [14]; and n=65 [23]), we found unbiased mean estimates of various sleep and HR variables, although the data generally exhibited wide LoA. In addition, we noticed problems in capturing the first REM sleep episodes. The naturalistic design of the study in a heterogeneous sample in terms of age and sex and regularly performing shift work increased the external validity and benefited our understanding of the Fitbit Charge 2’s performance in a minimally controlled home environment. Nevertheless, for the reliable use of consumer-grade sleep technology for clinical and research purposes, access to raw data, the use of open-source data analysis algorithms, more control of the data flow to blind users, and compliance with all regulatory aspects are indispensable. Furthermore, future validation studies should also be conducted in populations with sleep disorders, such as narcolepsy, who often present with S_on_ REM sleep episodes that appear particularly difficult to detect. Such studies can help identify the factors that determine the accuracy of Fitbit’s sleep and HR measures.

## Appendix

Multimedia Appendix 1 Supplemental figures and tables.

## Abbreviations

bpm: beats per minute
deep_d_: deep sleep duration
EBE: epoch-by-epoch
HR: heart rate
HR_10_: 10%-trimmed heart rate average
HRvar_10_: 10%-trimmed heart rate variance average
IBI: interbeat interval
light_d_: light sleep duration
LoA: limits of agreement
MCC: Matthews correlation coefficient
N1_on_: N1 sleep onset
N2_on_: N2 sleep onset
NPV: negative predictive value
PPV: positive predictive value
PSG: polysomnography
PSQI: Pittsburgh Sleep Quality Index
PSS-10: Perceived Stress Scale 10
QAVSD60: 60% quantile of the absolute value of the second derivative
REM: rapid eye movement
REM_d_: rapid eye movement sleep duration
REML: rapid eye movement sleep latency
rMEQ: Horne-Östberg Morningness-Eveningness Questionnaire-A Reduced Scale
S_off_: sleep offset
S_on_: sleep onset
TST: total sleep time
WASO: wakefulness after sleep onset
