# Supplementary material for: Validation of Fitbit Charge 2 Sleep and Heart Rate Estimates Against Polysomnographic Measures in Shift Workers: Naturalistic Study
Source: J Med Internet Res. 2021 Oct 5;23(10):e26476. doi: 10.2196/26476 (PMC8527385; doi:10.2196/26476)
Supplement: Multimedia Appendix 1 [file jmir_v23i10e26476_app1.docx]

**Validation of Fitbit Charge 2™ Sleep and Heart Rate Estimates against Polysomnographic Measures in Shift Workers: Naturalistic Study**

Benjamin Stucky^1,2*^; Ian Clark^1*^; Yasmine Azza^3,4,5^; Walter Karlen^2,6^; Peter Achermann^2,7^; Birgit Kleim^2,3,4^; Hans-Peter Landolt^1,2^

*^1^Institute of Pharmacology and Toxicology, University of Zurich, Zurich, Switzerland*

*^2^Sleep & Health Zurich, University Center of Competence, University of Zurich, Switzerland*

*^3^Department of Experimental Psychopathology and Psychotherapy, University of Zurich, Zurich, Switzerland*

*^4^Department of Psychiatry, Psychotherapy and Psychosomatics, University Hospital for Psychiatry, University of Zurich, Zurich, Switzerland*

*^5^Department of Psychiatry and Psychotherapy, Translational Psychiatry Unit, University of Lubeck, Lubeck, Germany*

*^6^Mobile Health Systems Lab, Department of Health Sciences and Technology, ETH Zurich, Zurich, Switzerland*

*^7^The Key Institute for Brain-Mind Research, Department of Psychiatry, Psychotherapy and Psychosomatics, University Hospital for Psychiatry, University of Zurich, Zurich, Switzerland*

*these authors contributed equally

**Supplemental Material**

**Address for correspondence:**Dr. Hans-Peter Landolt
Institute of Pharmacology & Toxicology
University of Zürich
Winterthurerstrasse 190
8057 Zürich, Switzerland
Phone: +41 44 635 59 53
Email: [landolt@pharma.uzh.ch](mailto:landolt@pharma.uzh.ch)


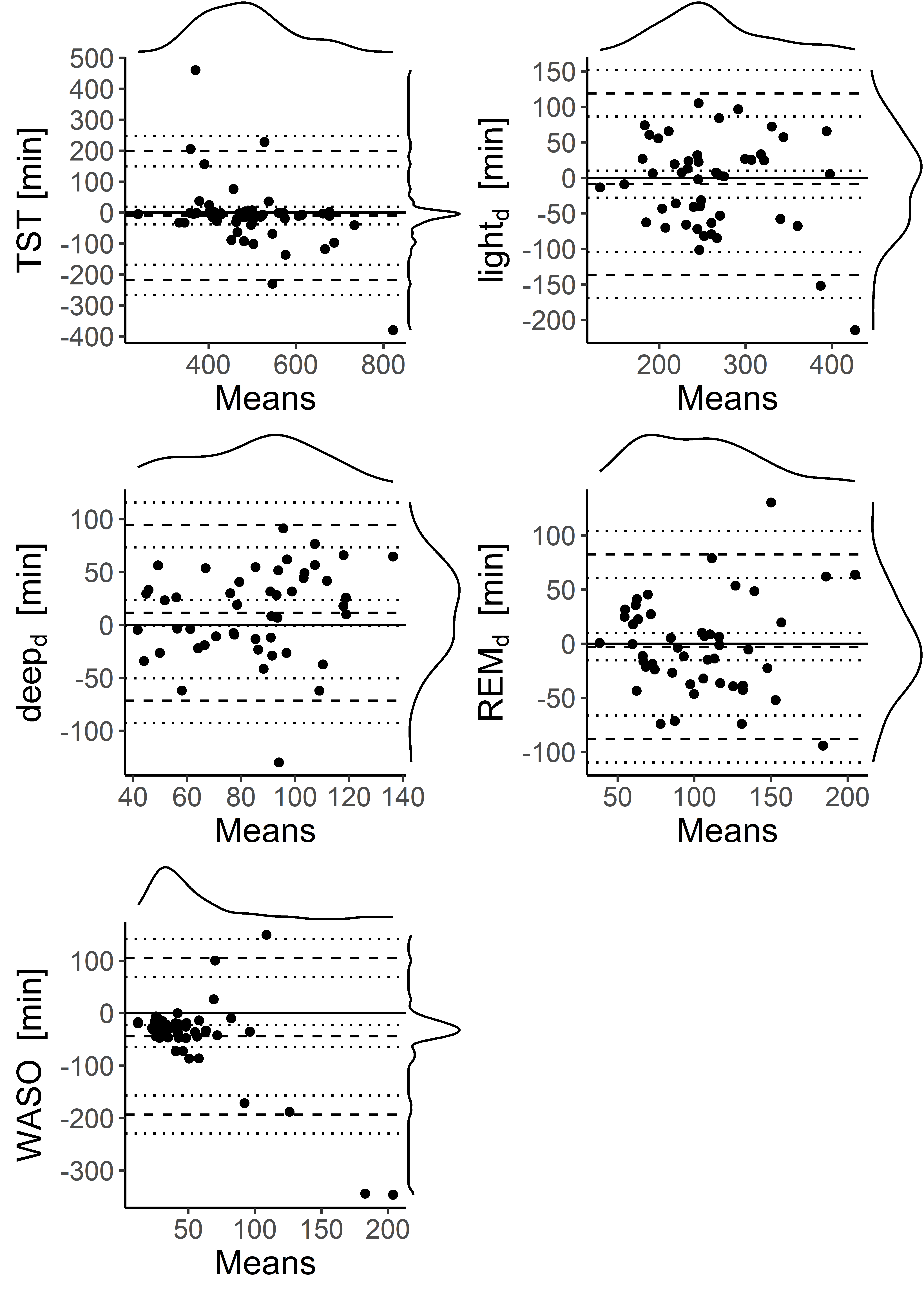
**Supplemental Figure S1:** Bland-Altman plots for the standard Fitbit variables are shown without adjusting for bordering wake episodes and without additional calculation of non-standard Fitbit variables like sleep onset, sleep offset and REM latency.

**Legend to the figure:** The dashed lines denote lower Limits of Agreement (LoA), bias and upper LoA. The dotted lines are the respective 95% confidence intervals of LoA. On the top and right of each panel the marginal densities are plotted. The x-axis displays the PSG variables, and the y-axis denotes the differences of the two devices (PSG - Fitbit). Total sleep time (TST), light sleep or N1 + N2 sleep duration respectively (light_d_) and deep sleep or N3 sleep duration (deep_d_) do not have a significant bias. Wake after sleep onset (WASO) display a significant deviation of the difference between the devices from 0. The results are very close to the boarding wake adjusted variables used in the main file. The wake adjustment in Fitbit especially improves the bias of total sleep time and wake after sleep onset.

**Supplemental Table S1:** Statistics accompanying the Bland-Altman plots in supplemental Figure S1 with the standard Fitbit variables.

| Variable | PSG-Fitbit | lower LoA | upper LoA | p-value |
| --- | --- | --- | --- | --- |
| TST [min] | -9.7 | -217.5 | 198.2 | 0.499 |
| REM_d_ [min] | -2.7 | -87.8 | 82.4 | 0.673 |
| Light_d_ [min] | -10.4 | -136.8 | 116.0 | 0.270 |
| Deep_d_ [min] | 11.2 | -72.9 | 95.2 | 0.078 |
| WASO [min] | -43.9 | -193.3 | 105.5 | 0.000 |

The results are very close to the adjusted Fitbit variables used in the main file. The adjustment in the main file especially improves the bias of total sleep time and wake after sleep onset.

**Supplemental Figure S2:** Bland-Altman plots for various sleep variables are shown with sleep onset defined as the first occurrence of N2 and with the variables adjusted for bordering wake epochs.


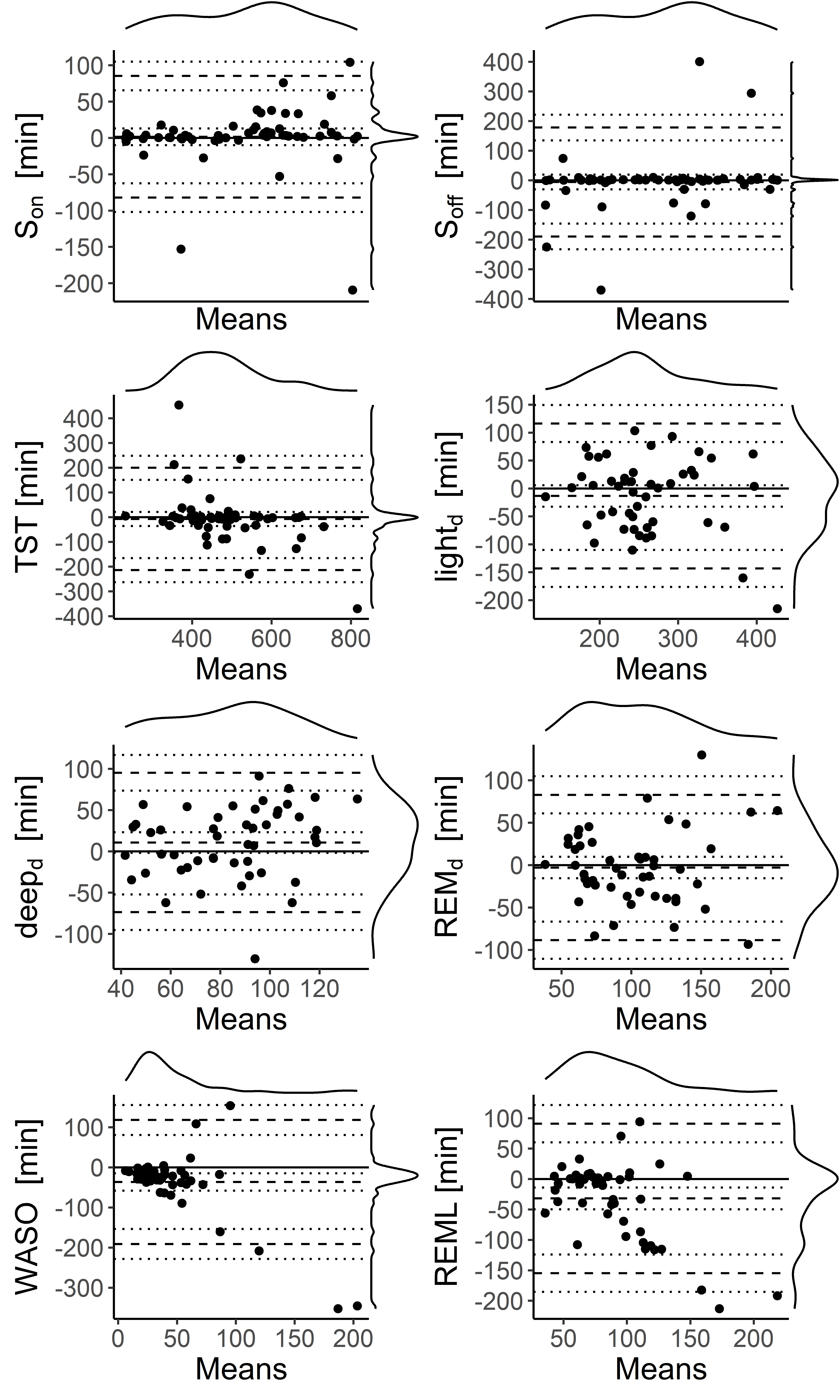


**Legend to the figure:** Bland-Altman plots for various sleep variables are shown with sleep onset defined as the first occurrence of N2 and with the variables adjusted for bordering wake epochs. The dashed lines denote lower Limits of Agreement (LoA), bias and upper LoA. The dotted lines are the respective 95% confidence intervals of LoA. On the top and right of each panel the marginal densities are plotted. The x-axis displays the PSG variables, and the y-axis denotes the differences of the two devices (PSG - Fitbit). N1 derived sleep onset is unbiased. Sleep offset (S_off_), total sleep time (TST), light sleep or N1 + N2 sleep duration respectively (light_d_), deep sleep or N3 sleep duration (deep_d_), and REM sleep duration (REM_d_) do not have significant bias. Wake after sleep onset (WASO) and REM sleep latency (REML) display a significant deviation of the difference between the devices from 0. Compared to Table 5 in the main file, which displays the N1 sleep onset results, the N2 sleep onset results are slightly more biased,

**Supplemental Table S2:** Statistics accompanying Bland-Altman plots in supplemental Figure S2.

|  | N2_on_ | | | |
| --- | --- | --- | --- | --- |
| Variable | PSG-Fitbit | lower LoA | upper LoA | p-value |
| S_on_ [min] | 6 | -62.9 | 74.9 | 0.209 |
| S_off_ [min] | -5.6 | -189.3 | 178.2 | 0.659 |
| TST [min] | -11.5 | -212.1 | 189.0 | 0.403 |
| REM_d_ [min] | -2.9 | -88.6 | 82.9 | 0.654 |
| Light_d_ [min] | -14.7 | -142.6 | 113.2 | 0.125 |
| Deep_d_ [min] | 10.5 | -74.8 | 95.9 | 0.100 |
| WASO [min] | -39.7 | -188.8 | 109.4 | 0.000 |
| REML [min] | -36.8 | -162.9 | 89.4 | 0.000 |

For sleep onset (S_on_), sleep offset (S_off_), total sleep time (TST), REM sleep duration (REM_d_), Fitbit light sleep or PSG N1+N2 sleep duration respectively (light_d_), Fitbit deep sleep or PSG N3 sleep duration respectively (deep_d_), wake after sleep onset (WASO) and REM sleep latency (REML). S_on_ and REML were calculated with N2 sleep onset criteria (N2_on_). The average difference between PSG and Fitbit measures the bias and can be found in the “PSG-Fitbit” column. The lower and upper LoA describe 1.96 times the standard deviation around the bias and can be found in the subsequent columns. In the last column the p-value for the paired t-test is reported, testing whether the bias is significantly different from 0. The various shades of green (darker colors corresponding to smaller *p*-values) highlight significant (p < 0.05) biases.
